# A Novel Bacterium Associated with Lymphadenitis in a Patient with Chronic Granulomatous Disease

**DOI:** 10.1371/journal.ppat.0020028

**Published:** 2006-04-14

**Authors:** David E Greenberg, Li Ding, Adrian M Zelazny, Frida Stock, Alexandra Wong, Victoria L Anderson, Georgina Miller, David E Kleiner, Allan R Tenorio, Lauren Brinster, David W Dorward, Patrick R Murray, Steven M Holland

**Affiliations:** 1 Laboratory of Clinical Infectious Diseases, National Institute of Allergy and Infectious Diseases, National Institutes of Health, Bethesda, Maryland, United States of America; 2 Microbiology Service, Department of Laboratory Medicine, W. G. Magnuson Clinical Center, National Institutes of Health, Bethesda, Maryland, United States of America; 3 Diagnostic and Research Services Branch, National Institutes of Health, Bethesda, Maryland, United States of America; 4 Laboratory of Pathology, National Cancer Institute, National Institutes of Health, Bethesda, Maryland, United States of America; 5 Section of Infectious Diseases, Department of Medicine, Rush Medical College, Chicago, Illinois, United States of America; 6 Microscopy Branch, Rocky Mountain Laboratories, National Institute of Allergy and Infectious Diseases, National Institutes of Health, Hamilton, Montana, United States of America; Institut Pasteur, France

## Abstract

Chronic granulomatous disease (CGD) is a rare inherited disease of the phagocyte NADPH oxidase system causing defective production of toxic oxygen metabolites, impaired bacterial and fungal killing, and recurrent life-threatening infections. We identified a novel gram-negative rod in excised lymph nodes from a patient with CGD. Gram-negative rods grew on charcoal-yeast extract, but conventional tests could not identify it. The best 50 matches of the 16S rRNA (using BLAST) were all members of the family Acetobacteraceae, with the closest match being Gluconobacter sacchari. Patient serum showed specific band recognition in whole lysate immunoblot. We used mouse models of CGD to determine whether this organism was a genuine CGD pathogen. Intraperitoneal injection of gp91^*phox*^^*−/−*^ (X-linked) and p47 ^*phox −/−*^ (autosomal recessive) mice with this bacterium led to larger burdens of organism recovered from knockout compared with wild-type mice. Knockout mouse lymph nodes had histopathology that was similar to that seen in our patient. We recovered organisms with 16S rRNA sequence identical to the patient's original isolate from the infected mice. We identified a novel gram-negative rod from a patient with CGD. To confirm its pathogenicity, we demonstrated specific immune reaction by high titer antibody, showed that it was able to cause similar disease when introduced into CGD, but not wild-type mice, and we recovered the same organism from pathologic lesions in these mice. Therefore, we have fulfilled Koch's postulates for a new pathogen. This is the first reported case of invasive human disease caused by any of the Acetobacteraceae. Polyphasic taxonomic analysis shows this organism to be a new genus and species for which we propose the name *Granulobacter bethesdensis.*

## Synopsis

As new bacteria continue to be discovered every year, it is inevitable that some of them will be found to cause human disease. The authors describe the isolation and characterization of a new bacterium, grown from a patient with chronic granulomatous disease (CGD). In this genetic disease, one of the main lines of defense against infection, the neutrophil, has a discrete defect in the generation of superoxide, leading to recurrent infections with a narrow spectrum of bacteria and fungi. This new organism was cultured from lymph nodes that had been inflamed for several months. To prove that this new bacterium was indeed a pathogen, Greenberg and colleagues measured specific antibody response in the patient: they inoculated CGD mice with this organism and reproduced the appearance of the human infection; they recovered the organism in pure growth from infected mouse spleens.

This new bacterium belongs to the family Acetobacteraceae, bacteria that are found widely in the environment. They have a variety of industrial uses, such as the production of vinegar, but have never been reported to cause invasive human disease. Disease-causing organisms remain to be discovered. The researchers outline some of the steps that can be taken to verify the pathogenicity of novel organisms.

## Introduction

Chronic granulomatous disease (CGD) is a rare inherited disease of the phagocyte NADPH oxidase system that leads to defective production of toxic oxygen metabolites and impaired killing of certain microbes [1]. Clinically, patients develop recurrent life-threatening infections with catalase-producing organisms as well as tissue granuloma formation [2]. The bacteria and fungi that commonly cause infection in CGD include: *Staphylococcus aureus, Serratia marcescens, Burkholderia cepacia, Nocardia* and *Aspergillus* species [3–7]. Rare infections with organisms only encountered in CGD, such as *Paecilomyces sp.* and *Trichosporon inkin,* suggest that patients with CGD have a unique susceptibility pattern [1]. However, when a specific microbiologic diagnosis cannot be made, patients are frequently treated with broad-spectrum antimicrobials that cover the most likely pathogens.

In the course of evaluation of fever and lymphadenitis in a patient with CGD, we identified a novel gram-negative rod. This organism belongs to a family of bacteria that has not been reported to cause invasive human disease. To prove that this organism was the cause of this patient's clinical syndrome, we utilized classical methods first described by Robert Koch in the late 19th century [8], supplemented with modern immunologic and genetic approaches. When faced with new potential pathogens, there remain situations in which Koch's postulates are as necessary to understand the etiology of infections as they were over a hundred years ago.

### Case Report

The patient was a 39-year-old man with X-linked chronic granulomatous disease (CGD) complicated by multiple episodes of S. aureus pneumonia and *Aspergillus* pneumonia, and a history of S. aureus liver abscesses that required drainage. He had remained healthy on trimethoprim-sulfamethoxazole alone for many years. In April 2003 he was hospitalized for fever and chills after returning from a trip to the Bahamas. Blood and urine cultures, cytomegalovirus serologies, cryptococcal antigen, and a transthoracic echocardiogram were not diagnostic. He had evidence of remote Epstein-Barr virus infection. A computed tomography scan of his chest showed new necrotic subcarinal and right hilar lymph nodes and two calcified granulomata in the right lower lobe. Inpatient intravenous vancomycin, ceftazidime, and voriconazole followed by outpatient oral voriconazole and trimethoprim-sulfamethoxazole were not curative. One month later, while on his outpatient regimen, repeat blood, sputum, and urine cultures were again negative. Cerebrospinal fluid examination was unremarkable. Gallium and Indium scans showed increased uptake in the right cervical and anterior mediastinal lymph nodes. Mediastinoscopy with biopsy of carinal lymph nodes showed caseating granulomatous inflammation. Calcofluor white, AFB (acid-fast bacillus), and gram stains were negative as were fungal and bacterial cultures. In addition, AFB cultures were negative after eight weeks. Because of three months of fever, chills, and 10-lb weight loss despite empiric therapy, the patient was referred to the National Institutes of Health.

**Figure 1:**
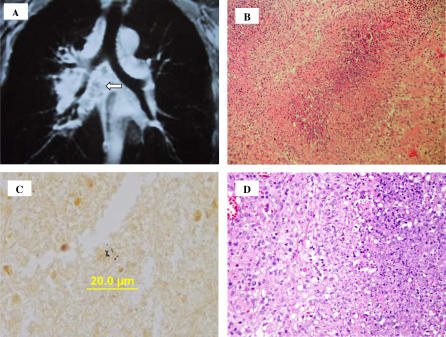
Clinical and Pathological Images of the Patient's Lymph Nodes (A) MRI of the chest showing adenopathy in the precarinal, subcarinal, and hilar regions. Central areas of diminished enhancement suggest necrosis (arrow). (B) Removed cervical lymph node showing necrotizing granulomatous lymphadenitis with abscess formation (pyogranuloma). (C) Warthin-Starry stain of the cervical lymph node (magnification 600×) showing coccobacillary organisms. (D) Higher magnification H&E of a necrotizing granuloma. There is an area of neutrophils and cellular debris on the right, bounded by a poorly defined layer of palisaded epithelioid histiocytes. Outside this layer is a mix of lymphocytes and histiocytes.

MRI of the chest showed mediastinal lymphadenopathy in the precarinal, subcarinal, and hilar regions with extensive necrosis (in Figure 1, see Figure 1A). Fevers, chills, fatigue, and night sweats persisted despite addition of azithromycin and moxifloxacin. Five months after symptom onset, he developed new painful left supraclavicular lymphadenopathy. Given the lack of response to empiric antimicrobials and the lack of microbiologic diagnosis, four involved left cervical and supraclavicular lymph nodes were removed. Pathology showed extensive necrotizing pyogranulomatous lymphadenitis with microabscess formation (Figure 1B and 1D). Fungal, gram, and AFB stains on sectioned lymph nodes were negative. A Warthin-Starry stain showed scant coccobacillary forms (Figure 1C). A novel gram-negative rod grew from three lymph node cultures on buffered charcoal yeast extract agar plates after four days of incubation.

The patient was initially treated in the fall of 2003 with doxycycline, meropenem, and a steroid taper. His remaining lymph nodes decreased in size over subsequent months. However, fever and enlarging lymphadenopathy returned in the fall of 2004. Since that time, he has required cervical lymph node excisions on four separate occasions. In May, June, and November of 2005, this new organism was again cultured from excised lymph nodes.

## Results

Gram-negative rods were isolated from three of the four cervical lymph node cultures, on buffered charcoal yeast extract plates (Remel, Lenexa, Kansas, United States) after four days incubation at 35 °C. The organism was a non-motile coccobacillus that was catalase-positive and oxidase-negative. Lysine and ornithine decarboxylases and arginine dihydrolase all were negative. Urease was positive; there was weak fermentation of glucose, and there was no acid production from lactose, mannitol, sucrose, maltose, or xylose. These results were confirmed at the Centers for Disease Control and Prevention (L. Helsel, personal communication). The API 20 NE (BioMerieux, Durham, North Carolina, United States) and RapID NH (Remel) commercial kits generated codes 0200000 and 1501, respectively, neither of which yielded an identification.

**Figure 2:**
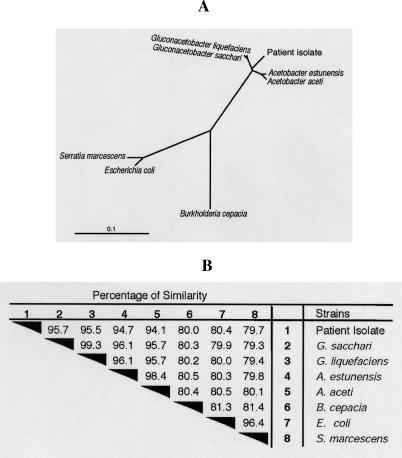
Novel Bacterium Groups in the Family Acetobacteraceae (A) Phylogenetic tree derived from the 16S rDNA sequence of the patient isolate, two *Gluconacetobacter* spp, two Acetobacter spp., and other gram-negative rods associated with CGD *(S. marcescens, B. cepacia)* and not associated with CGD *(Escherichia coli)*. This unrooted radial tree shows the patient isolate emerging as an independent branch that clustered together with members of the family Acetobacteraceae. The scale bar represents the number of substitutions per site. (B) Percentage of similarity of 16S rDNA sequences of the patient isolate, two Gluconacetobacter spp., two Acetobacter spp., and the gram-negative rods shown in (A).

In view of the failure of conventional tests to identify the organism and its fastidious growth characteristics, we sequenced the 16S rRNA gene and searched for related sequences in GenBank. The best 50 matches by BLAST search were all members of the family Acetobacteraceae, with the closest match being Gluconacetobacter sacchari (95.7% similarity) and Gluconacetobacter liquefaciens (95.5% similarity). The isolate from May of 2005 also had 16S sequencing performed and was identical to the original isolate. The 16S rDNA sequence similarity between the patient isolate and other commonly encountered pathogenic gram-negative rods was very low (~80%) (Figure 2).

Initial attempts to perform antibiotic susceptibility testing by microdilution and by a disk susceptibility test failed due to poor growth in broth and on Mueller-Hinton agar, respectively. Therefore, susceptibility testing was done on tryptic soy agar (TSA) sheep blood agar plates by the E-test. Minimum inhibitory concentrations (MICs) read after 48 h and 96 h of incubation at 35 °C showed the following results: ciprofloxacin (MIC > 32), azithromycin (MIC > 256), cefepime (MIC > 256), ceftriaxone (MIC = 16), meropenem (MIC > 32), and chloramphenicol (MIC > 256); amikacin showed an MIC = 32. At 48 h incubation, doxycycline showed MIC = 16 and trimethoprim-sulfamethoxazole showed MIC = 0.38; however, at 96 h of incubation, resistance to both drugs was observed.

The isolation of a previously unknown gram-negative rod from a patient with a rare immune disorder does not immediately prove causality. Contamination can occur during surgery or in the laboratory. To confirm the biological relevance of the isolated organism to our patient, we sought to determine whether this organism was immunologically and immunopathologically linked to his disease.

**Figure 3:**
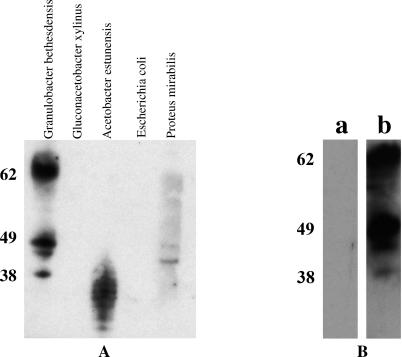
Immune Response to G. bethesdensis (A) An immunoblot showing the patient's plasma incubated with whole bacterial protein extracts of this new pathogen and other organisms as indicated (2 μg total protein per lane). Patient plasma was used at a 1:1000 dilution. (B) Patient's plasma from 1999 (a) and 2003 (b) incubated with whole bacterial protein extracts.

**Figure 4:**
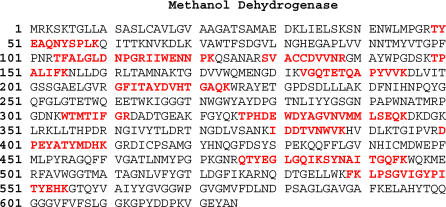
The Amino Acid Sequence of the Methanol Dehydrogenase Methanol dehydrogenase (69 kDa; EC 1.1.99.8) was identified as one of the peptides recognized by the patient plasma. Amino acids in red represent sequences that were matched during peptide mass fingerprinting.

To prove that this organism stimulated an immune response in our patient, whole bacterial lysates were exposed to unfractionated patient plasma in an immunoblot. Four distinct bands were seen at approximately 38 kDa, 45 kDa, 47 kDa, and 62 kDa. To confirm unique band recognition, neither related organisms *(Acetobacter estunensis, Gluconacetobacter xylinus)* nor unrelated organisms *(E. coli, Proteus mirabilis)* showed similar band patterns when incubated with the patient's serum (Figure 3A). In addition, sera stored from the patient years prior to the onset of his symptoms showed no reactivity on immunoblot (courtesy of Dr. Mary Dinauer, Figure 3B). To further characterize the recognized bands, two-dimensional gels of bacterial protein extracts were performed. Proteins detected with patient plasma by immunoblot were subjected to peptide mass fingerprinting (unpublished data). To further identify antigens being recognized on the two-dimensional gel immunoblots, protein extracts were run over a column coated with purified patient IgG. These two methods identified at least one of the proteins as a methanol dehydrogenase (69 kDa; EC 1.1.99.8). The recent completion of the genome sequence of this novel organism allowed us to specifically match this recognized peptide to this organism. The amino acid sequence of the recognized methanol dehydrogenase is shown in Figure 4. Studies to identify the other immunoreactive peptides are under way. Unique immunoblot protein recognition continues to be evident at dilutions of patient plasma of 1:100,000. Of interest, immunoblot patterns similar to those of this patient against this novel organism have been found in 3/19 patients with CGD and in 1/20 healthy individuals tested, respectively.

**Figure 5:**
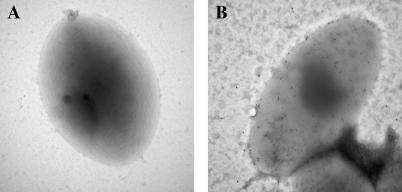
Immune Electron Microscopy of the New Bacterium Bacteria were adsorbed to Parlodion-coated grids and then probed with either normal plasma (A) or plasma from the CGD patient (B), labeled with anti-human IgG/colloidal gold, and negatively stained with uranyl acetate.

In addition to immunoblots, scanning immunoelectron microscopy and immunotransmission electron microscopy were performed. Immunotransmission electron microscopy images were taken after incubating colonies with either patient or normal plasma followed by gold-labeled anti-human IgG. Whereas bacteria incubated with plasma from normal humans failed to label with colloidal gold, those probed with plasma from the patient were clearly decorated with gold particles (Figure 5).

**Figure 6:**
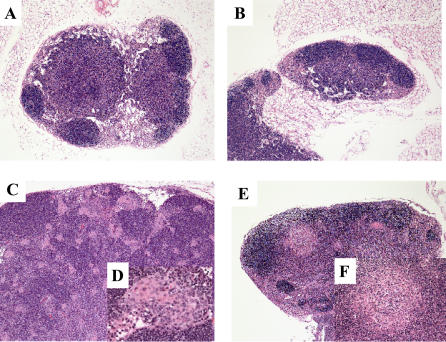
Histopathology of Mouse Lymph Nodes (A) Normal lymph node architecture of a gp91^*phox−/−*^ uninjected control mouse (magnification 5×). (B) Normal lymph node architecture of a gp91^*phox*^^*−/−*^ mouse after 10^7^ i.p. inoculation of Gluconobacter oxydans (magnification 5×). (C) Moderate lymphoid hyperplasia and presence of atypical macrophages in the lymph node of a gp91^*phox*^^*−/−*^ mouse after 10^6^ intraperitoneal inoculation of the novel organism (magnification 5×). (D) View of the epithelioid macrophages in the lymph node at 40×. (E) Lymphadenitis in the cervical lymph node of a gp91^*phox*^^*−/−*^ mouse after 10^7^ i.p. inoculation of the novel organism (magnification 5×). (F) View of the pyogranulomatous reaction in the lymph node at 20×. (A,B,E,F) are from mice that were sacrificed at nine days post-inoculation. (C and D) are from a mouse that was sacrificed at 76 days post-inoculation.

**Figure 7:**
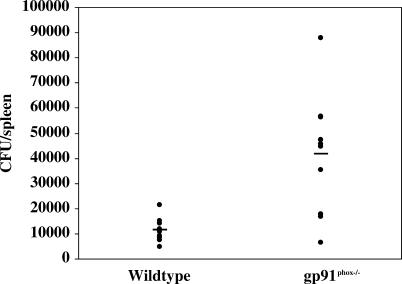
Graph Showing Organism Recovery from Spleen Homogenates at Nine Days Post-Inoculation The — mark shows the mean value. Mean CFU/spleen in the wild-type mice was 11,550, compared with 41,720 in the knockout animals (*p* < .001; two-tailed *t*-test).

Having confirmed that the patient had an immune response to the cultured organism, we sought to determine whether it showed pathogenicity in CGD. We used mouse models of CGD to determine whether this organism caused infection in the setting of CGD, and, if so, was it pathologically similar to that seen in our patient. gp91^*phox*^^*−/−*^ (X-linked) and p47^*phox*^^*−/−*^ (autosomal recessive) mice were injected with various amounts of organisms (10^2^ to 10^8^ CFU) i.p. and then organs and lymph nodes were harvested for culture and pathology at different time points. gp91^*phox*^^*−/−*^ and p47^*phox*^^*−/−*^ mice yielded histopathology similar to that seen in our patient's lymph nodes (Figure 6). In contrast, wild-type mice did not develop pyogranulomatous abscesses in the lung or lymph nodes, although a few demonstrated lymphoid hyperplasia. Splenomegaly was noted in both wild-type and CGD mice injected with the organism, but to a greater degree in the knockout mice. At all time points examined, organism recovery from spleen homogenates was greater in the CGD mice compared with wild-type mice. When 10^7^ CFU of bacteria were injected i.p. into ten wild-type and ten gp91^*phox*^^*−/−*^ mice, nine days post-inoculation the mean CFU/spleen in the wild-type mice was 11,550 compared with 41,720 in the gp91^*phox*^^*−/−*^ mice (Figure 7) (*p* < .001; two-tailed *t*-test). Gram stains, growth characteristics, and specific antibody recognition of the bacteria isolated from organ homogenates were similar to those of the patient's original culture. To prove that the pathologic organism recovered from the animals was the same as the one isolated from the patient, the 16S sequence was determined and was identical to the original isolate.

To determine if the pathology observed in CGD mice was unique to this new organism, or whether other members of the Acetobacteraceae could cause a similar disease, we injected six CGD and four wild-type mice with this new organism (10^7^ CFU) and another six CGD and five wild-type mice with another member of the Acetobacteraceae family, G. oxydans (ATCC 621H). Mice were sacrificed at nine days. All the mice, both wild-type and CGD, injected with the new bacterium showed pathologic changes in the lymph nodes ranging from moderate hyperplasia to the presence of plasmacytosis, atypical macrophages and lymphadenitis, although the changes in the wild-type mice were comparatively mild. In contrast, only 2/6 CGD mice and 2/5 wild-type mice injected with G. oxydans showed any pathologic changes, and these were limited to mild lymphoid hyperplasia and plasmacytosis. When taken together, the new bacterium caused more severe pathology when compared with another member of the Acetobacteraceae.

## Discussion

When we had identified a novel gram-negative rod from a CGD patient with fever and lymphadenitis, our first concern was to determine whether this organism was truly associated with his clinical syndrome. This question is critically important, insofar as this is a previously unrecognized organism without a body of literature to inform us as to its frequency or behavior.

Given that we have an animal model for CGD, we were able to utilize rules that were developed by Koch over one hundred years ago as he was trying to prove the etiology of tuberculosis. Although Koch's postulates cannot be applied in every situation, they are invaluable in the proof of causality. The isolation of this organism from our patient's diseased lymph nodes, its ability to cause similar disease when introduced into mouse models of CGD, the ability to isolate the same organism from these mice, and redemonstration of its identity prove that this novel bacterium in fact caused disease in our patient.

In addition to using an animal model to confirm pathogenicity of this organism in CGD, further evidence of its having infected this patient was demonstrated by his seroconversion from negative to high titer antibody. Screening of plasmas from other patients with CGD and people without it suggests that the bands at 38 kDa and 62 kDa may be characteristic of the human response to this organism. The recently completed genome sequence of this organism will prove valuable in determining the immunoreactive bands that are specific for this bacterium. When infected, both CGD and wild-type mice develop antibody. However, only CGD mice recapitulate the human immunopathology, confirming that this is a genuine pathogen in CGD.

This novel bacterium caused fever, lymphadenitis, and weight loss over almost six months in a patient with X-linked CGD, but was not fatal. Even at inocula as high as 10^8^, there were no deaths among the mice used in our challenge studies. We followed animals up to four months after infection; all mice cleared the infection spontaneously over time, as indicated by decreasing colony counts from organ homogenates. Interestingly, although pathologic changes were seen in the infected CGD mice, a few of the normal mice had low-level positive cultures from spleen homogenates for up to 76 days. Both CGD and normal mice produced antibody, as did our patient. The repeated isolations of this organism from our patient suggest that antibody production alone is not protective. It remains unclear whether the repeated isolation of this bacterium from our patient's diseased lymph nodes represents relapse or reinfection from as yet unidentified environmental reservoir(s). In either case, given its apparent low virulence, further investigation of this organism may shed light on the mechanisms of microbial killing in CGD. Comparing the genome of this organism to other available genomes of CGD pathogens may provide insight into common virulence properties.

Since the initial acceptance of this manuscript, we have isolated Granulobacter bethesdensis from two more individuals with CGD who presented with similar clinical syndromes. G. bethesdensis was confirmed with similar biochemical and molecular techniques, and the isolates were shown to be molecularly distinct.

These are the first reported cases of invasive human disease caused by any of the Acetobacteraceae. The family Acetobacteraceae comprises several genera including the genus *Gluconacetobacter* and *Acetobacter*. The closest 16S rRNA gene sequence match was to G. sacchari at 95.7%. However, strains that have a 16S rDNA sequence similarity below 97% are not considered the same species [9]. The sequence similarity of two species of *Gluconacetobacter* and two species of Acetobacter are shown in Figure 2. A polyphasic taxonomic approach shows that this organism belongs to a new genus and species in this family (unpublished data). Members of the genera *Gluconacetobacter, Acetobacter, Gluconobacter,* and *Acidomonas* [10] have been called the acetic acid bacteria, because they derive energy from the aerobic oxidation of ethanol to acetic acid. They have been cultured from fruits, fermented foods, plants, soil, and water [11–14], are utilized industrially in the production of vinegar, and are encountered during the fermentation of wine [15–18]. Given that this novel pathogen was isolated from a patient with CGD at the National Institutes of Health, we propose the name Granulobacter bethesdensis.

Patients with CGD are susceptible to infections caused by catalase-producing organisms. It is thought that organisms that produce catalase scavenge their own hydrogen peroxide and thus deprive the CGD phagocyte of the opportunity to use oxidative metabolites against it [1]. However, many organisms are catalase-positive and do not cause disease in CGD. In fact, the list of catalase-positive organisms that cause disease in CGD is quite narrow. This suggests that there are mechanisms beyond catalase that confer pathogenicity upon organisms that infect patients with CGD. It is hoped that the addition of a new organism to the CGD-specific infection list will allow further characterization of these virulence pathways. Many questions remain regarding the clinical epidemiology of this newly identified organism, such as its environmental reservoir and its seroprevalence in other disease states. Given that the Acetobacteraceae are ubiquitous environmental organisms, it is striking that they have never been reported to cause invasive disease, although a case of peritonitis associated with Asaia bogorensis in a patient with a peritoneal dialysis catheter has been recently reported [19]. The difficulty in culturing this organism from pathologic specimens, and the apparent seropositivity even among healthy people, suggest that there have been other encounters with this organism that have not been found previously. The clinical manifestations of this pathogen in other patients with CGD, and potentially in patients without CGD, are important subjects for future investigation.

## Materials and Methods

### Microbiology.

The growth of the isolate was assessed on various media under different incubation conditions. The organism grew best at 35°C on Acetobacter Medium [20] with the following modifications: glucose (50.0 g/L), CaCO_3_ (12.5 g/L), autolyzed yeast (5.0 g/L), agar (15.0 g/L). Colonies growing on this modified Acetobacter Medium developed a yellow color after 5 days. Good growth after 4–5 days of incubation was also obtained on buffered charcoal yeast extract agar and on TSA with sheep blood (Remel). Growth was not improved in a CO_2_ atmosphere.

Commercial biochemical kits, supplementary phenotypic tests, and sequencing were used to identify the isolate. Commercial kits used included the API 20 NE (BioMerieux) and the RapID NH (Remel). Additional phenotypic tests included oxidase, catalase, oxidative-fermentative medium with different sugars (OF medium King, Remel), lysine and ornithine decarboxylases, arginine dihydrolase, urease test (Rapid Urea Slant, Hardy Diagnostics, Santa Maria, California, United States), and motility. Susceptibility testing was performed on TSA with sheep blood by the E-test (Amersham Biosciences Biodisk, Piscataway, New Jersey, United States).

### Sequencing and phylogenetic analysis.

The full 16S rRNA gene was sequenced using the MicroSeq Full Gene 16S rRNA Bacterial Isolation Sequencing Kit (Applied Biosystems, Foster City, California, United States), according to the manufacturer's protocol, and sequences were analyzed using the 3100 Genetic Analyzer (Applied Biosystems). The Lasergene program (version 5.51; DNASTAR, Madison, Wisconsin, United States) was used for sequence assembly and alignment. The assembled 16S rDNA sequence from the patient isolate was compared with 16S rDNA sequences available in GenBank databases using the standard nucleotide–nucleotide basic local alignment search tool (BLASTn) program (National Center for Biotechnology Information, Bethesda, Maryland, United States). Multiple sequence alignments of the 16S rRNA gene sequences from the isolate and the closest GenBank matches were done by the CLUSTAL W method.

Phylogenetic analyses were performed with the PHYLIP version 3.5c package [21]. Distance matrices based on Kimura's two-parameter model were produced with DNADIST program, and a neighbor-joining tree constructed with the NEIGHBOR program. The resulting unrooted tree was depicted using the TreeView version 1.4 package [22].

### Immunology studies: Preparation of protein extracts, SDS gel electrophoresis, and immunoblot analysis.

Bacterial cells were pelleted by centrifugation and extracted with the Pierce (Rockford, Illinois, United States) bacterial protein extraction reagent. Protein concentrations were determined using the Pierce protein assay. 2μg of total protein per lane were electrophoresed on 10% SDS–PAGE, and the separated proteins transferred to PVDF membrane (Invitrogen Life Technologies, Carlsbad, California, United States). The membrane was blocked in TBS buffer containing 5% nonfat dried milk and 0.05% Tween-20 before incubation with human or mouse serum (1:100 to 1:100,000 dilution). After three washes, the membrane was incubated with horseradish peroxidase conjugated goat anti-human or goat anti-mouse IgG (1:10000 to 1:20000; Amersham Biosciences, Little Chalfont, United Kingdom). The blots were developed using the enhanced chemiluminescence kit (ECL Plus, Amersham Biosciences) as described by the manufacturer.

### Challenge studies: Mice.

C57BL/6 wild-type (Jackson Lab, Bar Harbor, Maine, United States) and gp91^*phox*^^*−/−*^ (Jackson Lab) and p47^*phox*^^*−/−*^ (Taconic, Hudson, New York, United States) mice extensively backcrossed to the C57BL/6 background [23,24] were maintained at the National Institute of Allergy and Infectious Diseases animal facility under specific pathogen-free conditions. All experiments were approved by the Institute's Animal Care and Use Committee. Mice were 4–8 mo old and sex-matched for each set of experiments.

### Mouse infection and sample collection.

The organism propagated from the patient's cervical lymph node cultures served as the source of the bacteria used in the mouse challenge studies. For these challenge studies, bacterial colonies growing on TSA with sheep blood were used to inoculate trypticase soy broth which was incubated for 10–14 days at 37 °C with 5% CO_2_. Bacteria were harvested by centrifugation and the bacterial pellet was washed twice in Hanks' balanced salt solution. Bacterial density was determined spectrophotometrically at 650 nm. Concentrations of bacteria from 2 × 10^8^ to 7 × 10^8^ CFU were serially diluted in Hanks' balanced salt solution and used to infect mice, and simultaneously plated on trypticase soy agar + 5% sheep blood plates (Remel) to quantify the inoculum. Mice were injected i.p. with 0.1 ml diluted bacteria. At 2, 9, 20, 76, and 126 days after challenge, blood was collected either retro-orbitally or from the tail into sterile tubes for culture and serum separation. Mice were then sacrificed by CO_2_ asphyxiation. Spleens and selected lungs were homogenized in 0.5 ml PBS. In one of the mouse experiments, spleens were homogenized in an amount of PBS proportional to the spleen's weight, so CFU/spleen could be determined. Samples of blood, spleen, or lung homogenates were serially diluted and inoculated in duplicate onto TSA with 5% sheep blood plates and incubated at 37 °C with 5% CO_2_ for 96–120 h, and colonies were enumerated. For histopathologic analysis, 3 ml of 10% phosphate-buffered formalin was injected into each lung; the inflated lungs and specified lymph nodes (cervical, axillary, and inguinal) were fixed in 10% phosphate-buffered formalin before section and staining.

## Supporting Information

### Accession Numbers

GenBank (http://www.ncbi.nlm.nih.gov/Genbank) accession numbers are: 16S rDNA sequence from the novel gram-negative rod G. bethesdensis (AY7889500); G. liquefaciens (X75617); and G. sacchari (AF127412).

## Abbreviations

CFU: colony-forming units
CGD: chronic granulomatous disease
MICs: minimum inhibitory concentrations
PBS: phosphate-buffered saline
TSA: tryptic soy agar
